# Dynamic multispectral NIR/SWIR for *in vivo* lymphovascular architectural and functional quantification

**DOI:** 10.1117/1.JBO.29.10.106001

**Published:** 2024-09-26

**Authors:** Christopher Hansen, Jaidip Jagtap, Abdul Parchur, Gayatri Sharma, Shayan Shafiee, Sayantan Sinha, Heather Himburg, Amit Joshi

**Affiliations:** a Medical College of Wisconsin, Department of Biomedical Engineering, Milwaukee, Wisconsin, United States; b Mayo Clinic, Department of Radiology, Rochester, Minnesota, United States; c Medical College of Wisconsin, Department of Radiation Oncology, Milwaukee, Wisconsin, United States; d Amity University, Amity Institute of Biotechnology, Centre for Medical Biotechnology, Noida, Uttar Pradesh, India

**Keywords:** lymphatics, shortwave infrared, quantum dots, indocyanine green, fluorescence

## Abstract

**Significance:**

Although the lymphatic system is the second largest circulatory system in the body, there are limited techniques available for characterizing lymphatic vessel function. We report shortwave-infrared (SWIR) imaging for minimally invasive *in vivo* quantification of lymphatic circulation with superior contrast and resolution compared with near-infrared first window imaging.

**Aim:**

We aim to study the lymphatic structure and function *in vivo* via SWIR fluorescence imaging.

**Approach:**

We evaluated subsurface lymphatic circulation in healthy, adult immunocompromised salt-sensitive Sprague–Dawley rats using two fluorescence imaging modalities: near-infrared first window (NIR-I, 700 to 900 nm) and SWIR (900 to 1800 nm) imaging. We also compared two fluorescent imaging probes: indocyanine green (ICG) and silver sulfide quantum dots (QDs) as SWIR lymphatic contrast agents following intradermal footpad delivery in these rats.

**Results:**

SWIR imaging exhibits reduced scattering and autofluorescence background relative to NIR-I imaging. SWIR imaging with ICG provides 1.7 times better resolution and sensitivity than NIR-I, and SWIR imaging with QDs provides nearly two times better resolution and sensitivity with enhanced vessel distinguishability. SWIR images thus provide a more accurate estimation of *in vivo* vessel size than conventional NIR-I images.

**Conclusions:**

SWIR imaging of silver sulfide QDs into the intradermal footpad injection provides superior image resolution compared with conventional imaging techniques using NIR-I imaging with ICG dye.

## 1 Introduction

Often described as the “forgotten system,”^1^ the lymphatic system is necessary for maintaining homeostasis and immune function. Lymph, which is composed primarily of soluble proteins, metabolites, and larger particulates, is formed in the periphery of cardiovascular circulation where interstitial fluid is taken up by lymphatic capillaries through gaps in the endothelial cells that make up the vessels.^2^ The lymphatic vessels are intrinsically contractile, and the lymph is moved in a peristaltic fashion with one-way valves to prevent backflow.^3^^,^^4^ Extrinsic pumping from skeletal muscle contraction also generates a pressure gradient, which drives lymph transport from the interstitium to larger vessels and lymph nodes.^5^ Transport of fluid containing interstitial antigens, chemokines, cytokines, and leukocytes to the lymph nodes is the primary immunological function of the lymphatic vessel network.^6^^–^^8^ Lymphatic dysfunction plays a critical role in many states of disease,^9^^,^^10^ and lymphatic vessels serve as the primary route for cancer metastasis.^11^ Lymph node metastasis has been correlated to poor clinical outcomes with lymphatic vessels enabling directional migration and metastatic seeding in the nodes.^12^

Despite these key functions of the lymphatic system, it remains poorly characterized relative to the cardiovascular system. A major challenge in characterizing the lymphatic system and disease-related lymphatic dysfunction is that the lymphatic network’s vessel structure and arrangement have high individual variability—the locations and branching are not as well conserved as blood vessels.^13^ This heterogeneity in lymphatic vessel architecture as well as their relatively small size makes them difficult to locate and therefore to quantitatively analyze.^14^ Previous *in vivo* imaging approaches including X-ray, fluorescence lymphangiography or lymphoscintigraphy, and photoacoustic imaging can visualize lymphatic structure and function but fall short of being able to quantify bulk lymph transport with high temporal resolution.^15^^–^^19^

Conventionally, fluorescence lymphangiography is performed using the imaging agent indocyanine green (ICG) and is imaged in the first near-infrared window (NIR-I, 700 to 900 nm). Shortwave-infrared (SWIR, 900 to 1800 nm) imaging or SWIR fluorescence imaging is a rapidly advancing technique for vascular imaging in rodent models. Here, we demonstrate *in vivo* that SWIR imaging provides substantially better spatial resolution relative to NIR-I-based lymphatic imaging. Such greater spatial resolution *in vivo* allows for more precise approximation and modeling of lymphatic spatial dynamics, which will improve upon current *in vivo* measurement methods of lymphatic function.

## 2 Methods

### 2.1 Animals

Adult female and male IL2Rγ salt-sensitive Sprague–Dawley (${\mathrm{SS}}^{\mathrm{IL}2R\gamma -}$) rats were bred and maintained at our institution. Animals were anesthetized during imaging with 2% of isoflurane maintenance in an oxygen flow of ∼1  L/min, and temperature was maintained throughout the experiment with a heating pad. All animals were imaged in a supinated position. All animal protocols were reviewed and approved by the Medical College of Wisconsin, Institutional Animal Care and Use Committee, where these experiments were performed. All rats recovered fully after fluorescence imaging.

### 2.2 Multispectral Imaging System

**Fig. 1:**
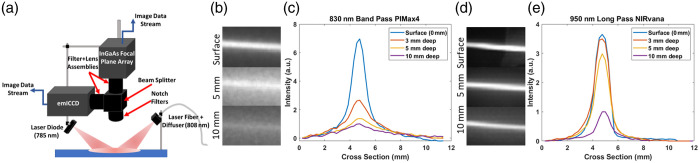
(a) Schematic of multispectral imaging system. (b) Capillary tube with 14  μM ICG excited with 785 nm with 830±10  nm emission in 1% liposyn at respective depths. (c) Cross section of ICG-filled capillary tube normalized to 10 mm depth in liposyn. (d) Capillary tube with 14  μM ICG excited at 808 nm with 980 nm long pass emission. (e) Cross section of ICG-filled capillary tube normalized to 10 mm depth in liposyn.

A combination of 785 nm (Thorlabs, L785P200, Newton, New Jersey, United States) and 808 nm (Diomed, D15 Plus, Cambridge, United Kingdom) laser light to excite the ICG and QDs, respectively, illuminated the entire animal body [Fig. 1(a)]. The imaging system comprised a 16-bit deep cooled intensifier electron-multiplying charge-coupled device (emICCD) camera (Princeton Instruments, PI-MAX4, Trenton, New Jersey, United States, 512×512  pixels, 400 to 900 nm sensitivity) and a 16-bit deep cooled InGaAs sensor focal plane array camera (Princeton Instruments, NIRvana 640ST, 512×640  pixels, 900 to 1700 nm sensitivity) in combination with a fused silica broadband beam splitter (Thorlabs, BSW30) to direct signal from the same field of view to both cameras. A combination of 785±5  nm (SuperNotch-Plus™, HSPF-785.0-2.0, Kaiser Optical System, Ann Arbor, Michigan, United States) and 808±20  nm (Semrock, StopLine^®^, NF03-808E-50) notch filters were used sequentially, pre-beam splitter; an 830±10  nm bandpass filter (830FS10-25, Andover, Salem, New Hampshire, United States) and 980 nm long pass filter (Semrock, EdgeBasic™, BLP01-980R-50, Rochester, New York, United States) were used post-beam splitter on the NIR and SWIR beam paths, respectively. For SWIR-only imaging, the NIRvana was used with the 808 nm laser and appropriate filters with no beam splitter.

### 2.3 Imaging Agents

Imaging agents were administered in a single bolus of 20  μL intradermally into the interdigital space of the rat hindfoot. ICG was administered at a concentration of 50 to 100  μM. QDs were synthesized via viscosity-mediated synthesis as described by Tang et al.^20^ QD emission spectra are governed by the size of the particles and can be modulated by increasing or decreasing the synthesis reaction time. Eight-nm QDs fluoresce around 1100 nm upon 808 nm excitation. PEGylated quantum dots (QDs) are grossly different than ICG in physical characteristics and thus exhibit different pharmacokinetics. After PEGylation (500  g/mol), the hydrodynamic radius of QDs was ∼60  nm with a zeta potential in the interval [−5,−10]  mV. QDs were administered with a concentration of ∼37.5  μg/mL in equal volumes to ICG and imaged using a custom dual-camera multispectral imaging system in both NIR and SWIR. Animals imaged with ICG were also imaged with PEGylated QDs after the ICG had cleared ∼2 weeks later. As a true measure of the lymphatic vessel diameter, Evan’s blue (EB) dye (5% in 25  μL Hank’s balanced salt solution) was injected in the interdigital foot pad of anesthetized rats following fluorescence imaging.^21^ Following euthanasia, ∼15  min after injection, the skin was dissected to reveal the dermal lymphatics, and RGB images of the EB-stained vessels on the underside of the skin were taken. To mimic the scattering properties of tissue, a 1% liposyn emulsion in PBS was used as a tissue phantom with a capillary tube serving as the vessel. The absorption spectra of tissue are relatively similar in both the NIR-I and SWIR, so no absorber was added to the liposyn emulsion.^22^

### 2.4 Image Processing and Derivation of Functional Parameters

Image streams were acquired using LightField software (Princeton Instruments), and image processing and data analysis were performed using MATLAB (Mathworks, 2023b) software. The MATLAB function “findpeaks” was used to identify and measure peaks and peak prominences in the vessel cross sections and identify peaks in the time series with empirically tuned parameters. For the purposes of comparison of the NIR versus SWIR imaging, vessel distinguishability was defined as the sum of the peak prominences in normalized, background-subtracted images from the same vessels in each camera frame divided by the number of vessels per centimeter of the line drawn, e.g.,

$$\text{Vessel Distinguishability}=\sum (\text{Peak prominences})/(\frac{\text{vessels}}{\mathrm{cm}}),$$ (1)

and the resolution of the images was defined as the full width at half maximum of the vessel peak.

To simplify the task of finding peaks corresponding to lymph transport in the time series, images were “Z-normalized.” The Z-normalization process removes the bias in identifying peaks and calculating transport velocity. Image processing for “Z-normalization” in MATLAB includes (1) segment image stack into pixels of interest i.e., mask based on hand-drawn regions of interest (ROI) or pixel intensity. (2) Convert 3D stack to 2D array in pixel X time format and Z-normalize each row by subtracting the row mean and dividing by standard deviation. (3) Wavelet denoise each row (pixel time series) using the “wdenoise” function with eighth-level symlet wavelets, empirical Bayesian denoising method with median thresholding, and a level independent noise estimation. (4) Subtract a wavelet reconstruction (using “waverec” with the wavelet scaling factors from the denoising output) from the denoised row and square the difference to remove negative values. (5) Convert 2D matrix back to 3D image stack. To compute lymph bolus transport velocity after Z-normalization, the time difference between a bolus (as indicated by peaks in a time series from an ROI on a vessel) at two points on the same vessel was calculated, and the distance the bolus traveled along the vessel between those points was measured. The EB vessel width was measured using ImageJ.

## 3 Results

### 3.1 Validation of Multispectral Imaging System

Comparison of NIR-I and SWIR imaging of ICG phantoms in a tissue-like scattering medium (1% liposyn emulsion) showed a relative increase in sensitivity and spatial resolution owing to lower scattering in the SWIR imaging regime [Figs. 1(b)–1(e)]. These differences in the visualization of the capillary fluorescence phantom were particularly evident at the 10 mm depth.

### 3.2 Determination of Lymphatic Vessel Cross Section Using NIR-I and SWIR Imaging

**Fig. 2:**
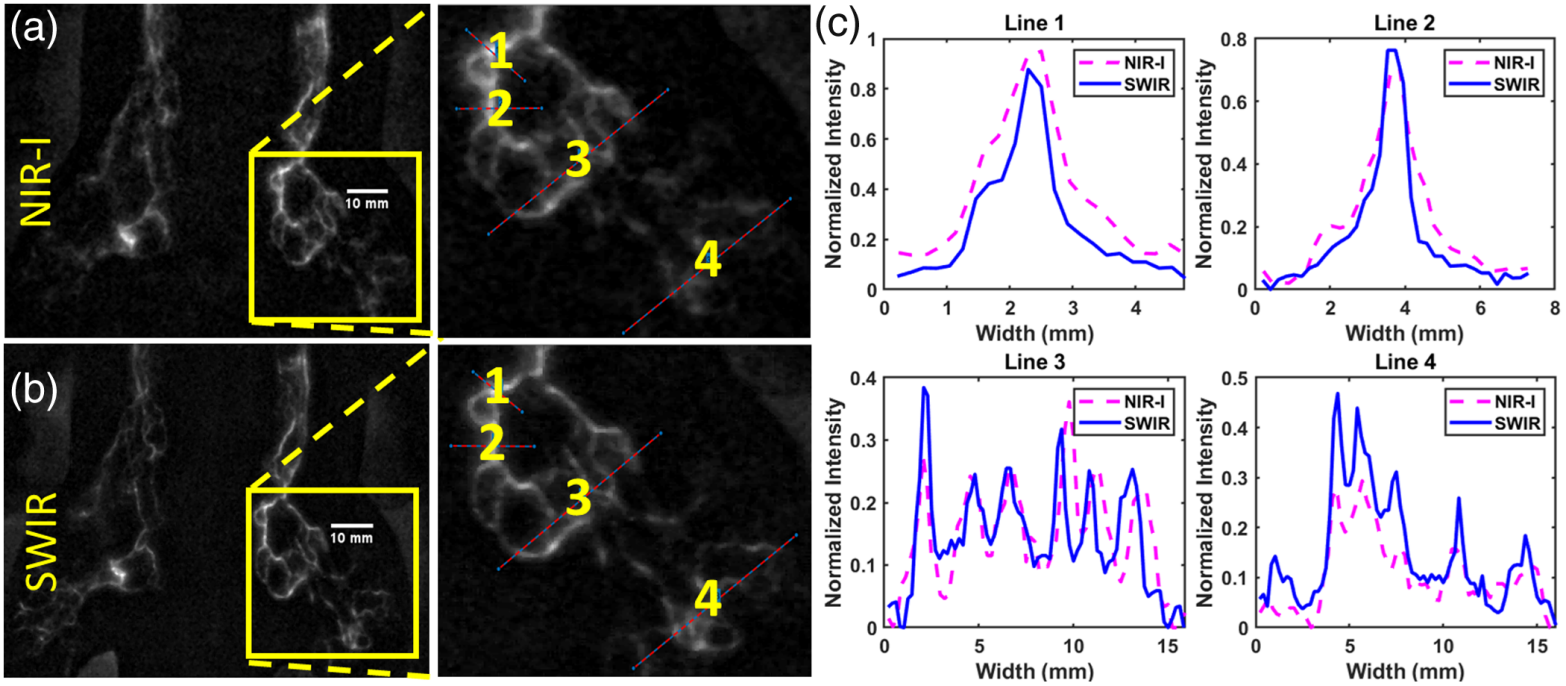
(a) Hindlimb and lower abdominal lymphatics in a supinated rat after intradermal ICG injection in the footpad with 830±10  nm emission with magnified inset. (b) Hindlimb lymphatics in the same rat at 808 nm with 980 nm long pass emission with magnified inset. (c) Maximum normalized vessel cross sections in respective spectral bands (Video 1, mp4, 2.09 MB [URL: https://doi.org/10.1117/1.JBO.29.10.106001.s1]).

We observed quantitative differences between NIR and SWIR ICG fluorescence images in the lymphatics following intradermal footpad injection (Table 1). As was expected from the phantom experiments, SWIR imaging showed superior spatial resolution *in vivo* of both small and large vessels using ICG as contrast [Figs. 2(a)–2(c)]. SWIR imaging with ICG provided up to 1.7 times the vessel distinguishability and up to 1.7 times the resolution. In addition, there was less excitation contamination in SWIR emission images due to the wide spectral separation in excitation and emission spectra. As the InGaAs sensor is not sensitive to 785 or 808 nm wavelengths, it provides an additional layer of long-pass filtering.

**Table 1:** Data from Fig. 2(c) showing sensitivity and resolution. Averages with standard deviation are reported for plots with more than one peak.

	Plot 1	Plot 2	Plot 3	Plot 4	Average
SWIR vessel distinguishability	0.83	0.75	0.26	0.31	0.54 ± 0.29
NIR-I vessel distinguishability	0.81	0.65	0.15	0.30	0.48 ± 0.30
Vessel distinguishability ratio (SWIR /NIR-I)	1.02	1.16	1.73	1.04	1.24 ± 0.33
SWIR resolution (mm)	0.76	0.85	0.94 ± 0.72	0.81 ± 0.23	0.84 ± 0.08
NIR-I resolution (mm)	1.26	1.35	1.12 ± 0.88	1.02 ± 0.24	1.19 ± 0.15
Resolution ratio (SWIR/NIR-I)	0.60	0.63	0.97 ± 0.29	0.88 ± 0.20	0.77 ± 0.18

### 3.3 Determination of Transport Velocity and Estimating Flow

**Fig. 3:**
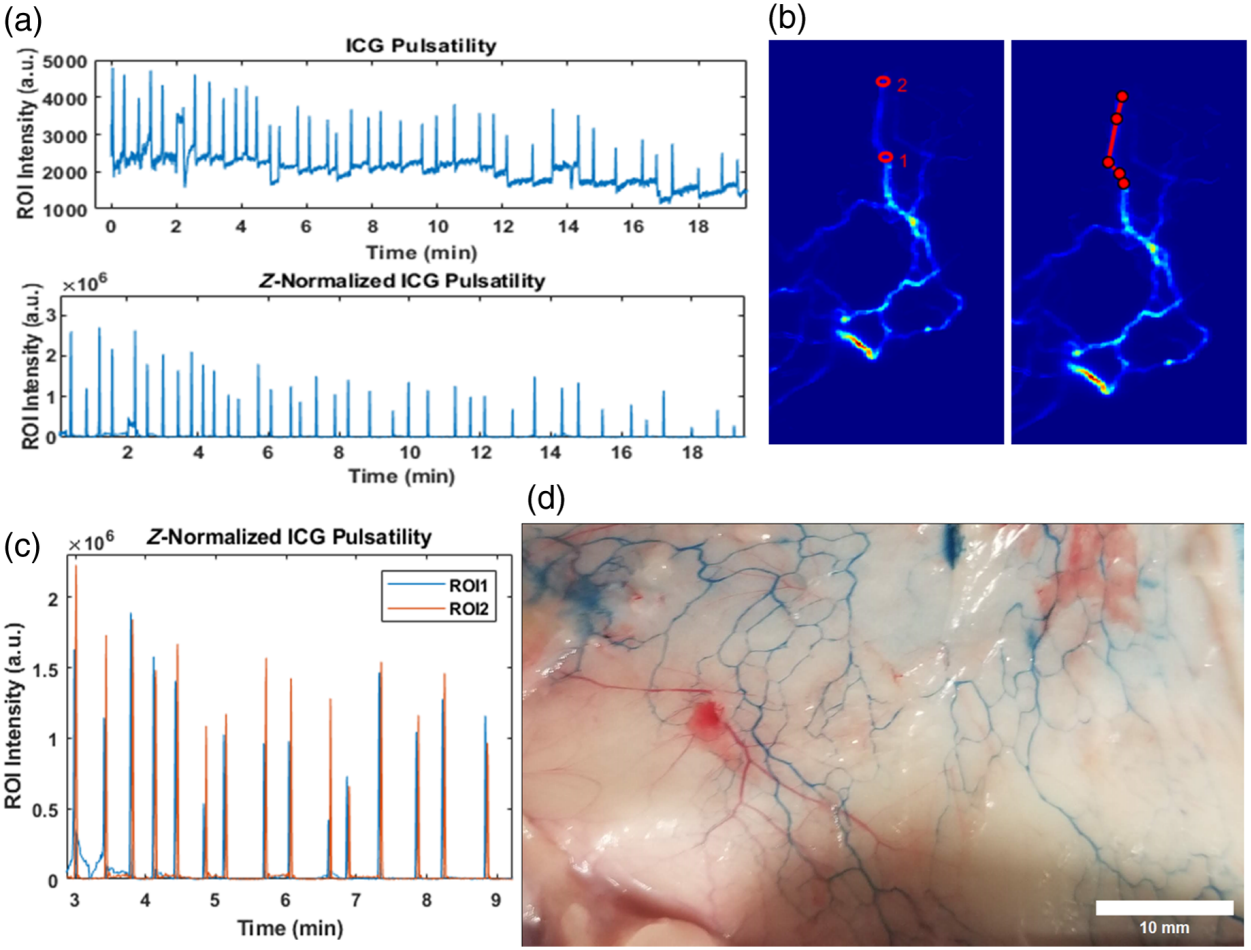
(a) Unnormalized and Z-normalized time series from a circular ROI drawn on a lymphatic vessel. (b) ROIs (left) and the line trace on the vessel (right) for computing lymph bolus transport velocity. (c) Z-normalized peaks for ROIS 1 (blue) and 2 (orange) in panel (b) used to measure time in transport velocity. (d) Subdermal lymphatics visualization following Evan’s blue injection used for true vessel diameter.

Distinct peaks indicating each bolus of lymph (Video 1) transiting a region of interest drawn on a lymphatic vessel can be observed following Z-normalization and wavelet denoising. With two ROIs drawn on the length of a vessel, the peaks in the time series—corresponding to boluses of transiting lymph—at each vessel segment can be used to determine the time it took for the bolus to transit the length of the vessel and subsequently the transport velocity [Figs. 3(a)–3(c)]. QDs and ICG both showed similar transport velocities of 6.39±2.40 and 7.06±1.58  mm/s, respectively (n=4 rats each), in the vessels visualized on the abdominal region. EB injections in the footpad allowed for hindlimb/abdominal subdermal lymphatics to be visualized upon dissection of the skin. The true physical size could then be measured and compared with the diameter of the vessels measured from fluorescence data using the same techniques as Fig. 2. The average diameter of vessels measured via EB was 0.22±0.04  mm (n=3 rats), whereas the same vessel measured via SWIR fluorescence was 1.10±0.38  mm (n=3 rats). Thus, the SWIR vessel cross section is five times greater due to multiple scattering of light through the skin than the true physical vessel measured by EB after dissection.

### 3.4 Comparison of QD and ICG

**Fig. 4:**
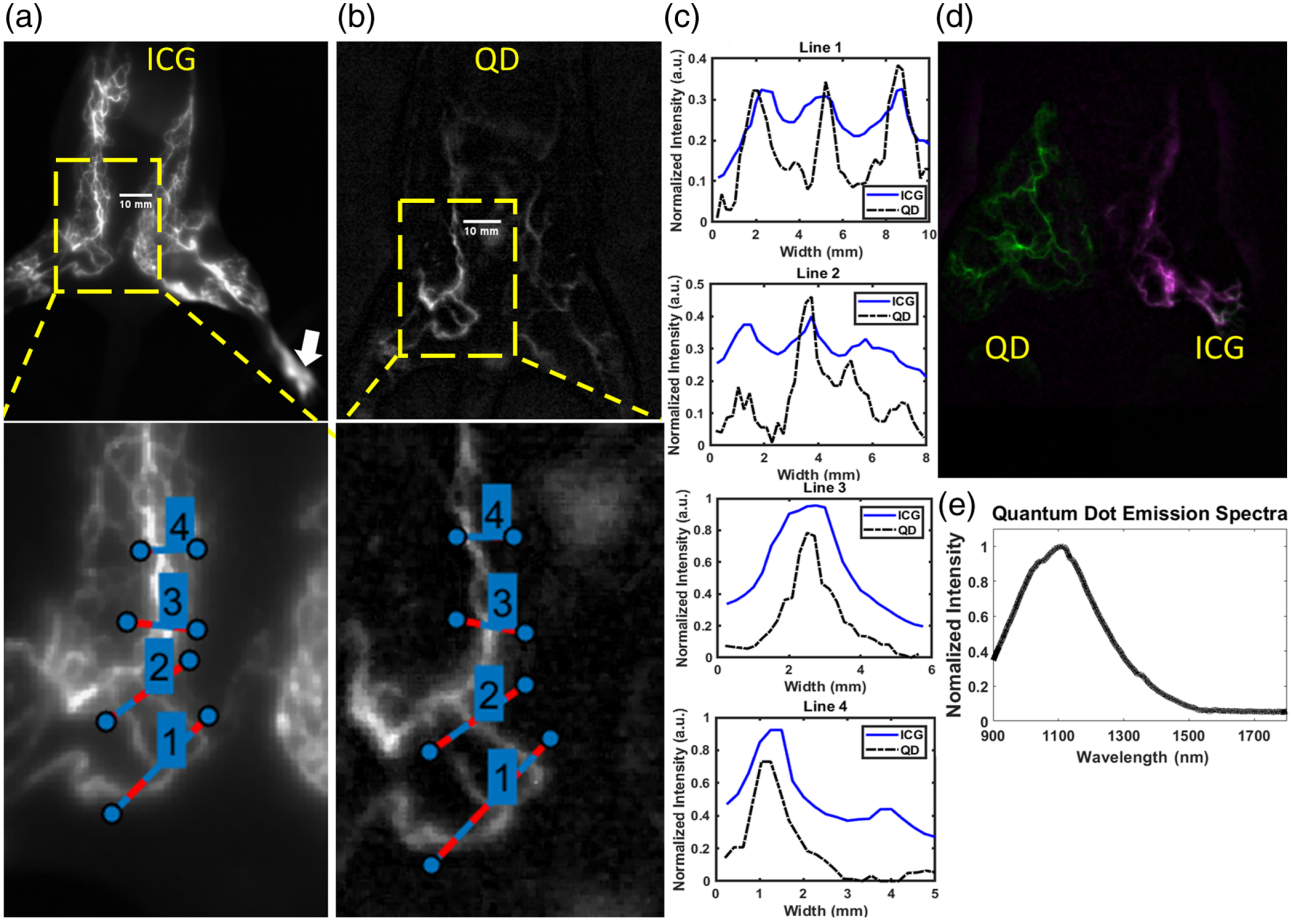
(a) Hindlimb and lower abdominal lymphatics after intradermal ICG injection in footpad (white arrow) with 980 nm long pass emission with magnified inset. (b) Hindlimb and lower abdominal lymphatics after intradermal QD injection in the footpad with 980 nm long pass emission with magnified inset. (c) Normalized vessel cross sections with respective SWIR contrast agents. (d) Co-registered images of simultaneous SWIR and NIR lymphatic images with NIR in purple and SWIR in green with overlapping signals appearing in white. QDs were administered to the supinated rat’s right hindlimb and ICG to the left. (e) Emission spectra of QDs.

ICG and QDs were injected in the same vessels of the same animals 13 days apart to allow for time for clearance of previous contrast administration. These images indicated that ICG-tagged vessels had a higher absolute intensity than the QDs but had an inferior signal-to-background ratio (Table 2) due to a higher background even after both images underwent pre-contrast background subtraction. The emission peak for ${\mathrm{Ag}}_{2}S$ QDs is red-shifted compared with ICG and has a tighter emission window (Fig. S1 in the Supplementary Material). This should result in sharper resolution of QD-SWIR imaging compared with SWIR imaging with ICG which was borne out by the results in Figs. 4(a)–4(c) for which both ICG and QD emissions were imaged with a 980 nm long-pass filter. Although the purely SWIR-emitting QDs show sharper spatial resolution due to reduced scattering, they are not as bright as ICG in the lymphatic vessels. Conjugation of thiol-terminated PEG to the carboxylic acid groups on the surface of the QDs created a more neutral zeta potential of approximately −7  mV from the −20  mV of the acid coating, which likely resulted in a faster clearance from lymphatics compared with ICG; consequently smaller lymphatics can show up as brighter for ICG imaging, as shown in Figs. 4(a) and 4(b).^23^ SWIR imaging with QDs provided up to 2.5 times the vessel distinguishability, dependent on the vessel spacing, and up to 1.7 times the resolution (Table 2). This could be due to the shorter SWIR emission wavelength of ICG—leading to higher scattering. Co-registration of NIR (830 nm BP) and SWIR (980 nm LP) images with concurrent ICG and ${\mathrm{Ag}}_{2}S$ QDs injections in opposite limbs shows that higher spatial resolution and lower background are possible with ${\mathrm{Ag}}_{2}S$ QDs [Fig. 4(d)]. A peak between 1100 and 1200 nm is expected with ${\mathrm{Ag}}_{2}S$ QDs of this size [Fig. 4(e)]. The rats injected with QDs did not suffer any apparent toxicity and cleared the residual QDs from the injection sites after approximately a week’s time.

**Table 2:** Data from Fig. 4(c) showing sensitivity and resolution. Averages with standard deviation are reported for plots with more than one peak. The resolution ratio was not calculated for dissimilar numbers of peaks.

	Plot 1	Plot 2	Plot 3	Plot 4	Average
SWIR QD vessel distinguishability	0.26	0.12	0.73	0.59	0.42 ± 0.28
SWIR ICG vessel distinguishability	0.10	0.12	0.62	0.50	0.34 ± 0.26
Vessel distinguishability ratio (QD/ICG)	2.49	0.98	1.18	1.18	1.46 ± 0.69
SWIR QD resolution (mm)	1.01 ± 0.20	0.58 ± 0.27	1.04	0.79	0.86 ± 0.21
SWIR ICG resolution (mm)	1.08 ± 0.12	0.75 ± 0.08	1.76	0.81	1.10 ± 0.46
Resolution ratio (QD/ICG)	0.94 ± 0.28	N/A	0.59	0.97	0.83 ± 0.21

## 4 Discussion

Historically, lymphatic vessel properties were characterized *ex vivo* using excised, perfused vessels placed on a fine wire myograph.^24^^,^^25^ This technique yields a time series of lymphatic vessel contractility, which can be used to examine vasodilators, genetics, and transmural pressure gradients on the function of lymphatic contractility *ex vivo*.^26^^,^^27^ Fluorescence imaging *in vivo* visualizes lymphatic pumping in real time—the intensity, duration, and frequency of these pulses are directly correlated to how much fluid the lymphatics are moving back into circulation, an important distinction compared with myography, in which the contractions do not necessarily result in functional peristaltic fluid transport.^28^^–^^31^ The main limitations of *in vivo* imaging are the difficulty of administering physiologically active compounds specifically into the vessel and the challenge of discriminating the potential impact on visualized lymphatic contractility due to interactions between the contrast agent and the lymphatics themselves.^32^^,^^33^

Near-infrared optical fluorescence in the 700 to 900 nm tissue transparency window has been used widely for *in vivo* imaging of small animal models in biomedical research and recently has begun to attract more attention for clinical use in image-guided procedures.^34^^–^^37^ Fluorescence optical imaging offers several advantages: it is highly sensitive—using nano to micromolar contrast doses, is relatively low cost, utilizes nonionizing radiation, offers high temporal resolution, and has a relatively high throughput relative to other macroscopic imaging modalities.^38^^–^^40^ It does, however, suffer one major drawback—as tissue depth increases, the signal-to-noise ratio (SNR) and spatial resolution suffer—precluding its use in most clinical applications.^41^ NIR-based imaging can interrogate centimeters deep via multiple scattering through most tissues owing to relatively low absorption and autofluorescence at these wavelengths.^42^ The SWIR spectrum has the additional advantages of much lower autofluorescence, the ability to use fluorescent probes with a large Stokes shift, and further reduced scattering, allowing for higher resolution; however, it is limited by a scarcity of fluorescent probes and imaging sensors.^22^^,^^43^

Many choices exist for fluorescent probes in the NIR-I, but only ICG and methylene blue (MB) are currently FDA-approved.^15^ The longer emission wavelength of ICG benefits from lower absorption and decreased autofluorescence yielding a higher signal-to-background ratio.^44^ The strong emission of ICG in the SWIR makes it the only SWIR probe that is FDA-approved.^22^ No SWIR-only emitting probes are currently FDA-approved, and the group mostly comprises low bandgap semiconductors such as QDs and single-walled carbon nanotubes, which need to be coated to make them biocompatible; however, at least one small molecule has been described.^45^^–^^48^ The combined administration of widely spectrally separated NIR and SWIR fluorescing probes to lymphovascular circulation can enable simultaneous investigation of the systemic function, pathology, and vascular phenotypes as well as the biodistribution and pharmacokinetics without spectral crosstalk.^49^^–^^52^ Although the lymphatic uptake of SWIR emitting QDs has been demonstrated, no quantitative functional lymphatic imaging has been reported.^53^ Biodistribution, toxicology, and pharmacokinetics of ${\mathrm{Ag}}_{2}S$ QDs have shown mixed toxicity results dependent on dose *in vitro.*^20^ Limited *in vivo* studies have shown no appreciable toxicity with fecal clearance through the reticuloendothelial system via the bile in rodent systems.^54^^,^^55^

Infrared fluorescence imaging data have been used extensively to validate mechanistic mathematical models of physiologic processes, particularly vascular function.^56^^–^^60^ Mathematical models of physiologic processes can generally be split into two classifications: simpler, lumped parameter models described by ordinary differential equations and continuum models described in higher dimensions by partial differential equations—models of both types exist for lymphatics.^61^^–^^65^ Continuum models greatly benefit from the inclusion of structural data.^66^ High-fidelity fluid dynamics models of blood flow throughout the cardiovascular system exist due to the vast amount of structural data on the system.^67^ With the high-resolution images that SWIR lymphatic imaging provides, equivalent models can be created for the lymphatic system.^44^^,^^68^ Dynamic fluorescence imaging at video rates creates large amounts of data, making the image time series an ideal candidate for machine learning and computer vision applications.^69^

The present study shows that SWIR imaging of the lymphatic network with silver sulfide QDs enables high-resolution imaging of lymphatic vessels and the quantification of parameters necessary to characterize lymphatic circulation such as flow rates and valve function. Greater spatial resolution *in vivo* with SWIR allows for more precise approximation and modeling of lymphatic spatial dynamics, which will improve upon the current approximation of lymphatic function. Higher spatial resolution with greater SNR is also more favorable for computer vision vessel segmentation algorithms yielding high-precision vessel networks from which time series data can be extracted and used in machine and deep learning algorithms. The lymphatic pharmacokinetics of pegylated QDs are also unexplored, and the biologic fates of these particles when administered via intradermal injection remain to be seen. The pure SWIR emission spectra, small size, and neutral charge of these particles make them ideal candidates for the study of lymphatic clearance because of the high SNR imaging that they enable and low binding to the vessel walls as compared with ICG. The high-resolution, dynamic imaging techniques that quantify lymphatic function in this project are applicable to any number of vascular disease states, such as hypertension and cancer.

## 5 Conclusion

We have demonstrated the use of a dual NIR and SWIR fluorescence imaging system in rats. This system can be adapted for a variety of *in vivo* imaging applications aside from lymphatics. We demonstrated that the use of ${\mathrm{Ag}}_{2}S$-based QDs as SWIR probes can result in nearly threefold higher sensitivity and up to 1.7 times higher spatial resolution in identifying lymphatic vessels. This increased sensitivity and resolution can pick up deeper and smaller subsurface lymphatics that can be missed by conventional ICG-based NIR fluorescence imaging. Thus, SWIR imaging with ${\mathrm{Ag}}_{2}S$ QDs can be a sensitive tool for probing lymph-angiogenesis in cancer and other pathology involving lymphatic remodeling and dysfunction. In addition, we have demonstrated that SWIR dynamic fluorescence imaging can be used to quantify lymphatic transport *in vivo*.

## Supplementary Material





## Data Availability

Data and code are available from the authors upon reasonable request.
