# Supplementary material for: Dynamic multispectral NIR/SWIR for in vivo lymphovascular architectural and functional quantification
Source: J Biomed Opt. 2024 Sep 26;29(10):106001. doi: 10.1117/1.JBO.29.10.106001 (PMC11425400; doi:10.1117/1.JBO.29.10.106001)
Supplement: Supplementary file 1 [file JBO_029_106001_SD001.pdf]

Supplementary Data: Absorption and NIR-2 emission spectra of ICG and Ag_2S Quantum dots

All UV-visible absorption studies were carried out for the liquid samples in a Cole Palmer made Jenway 6405 model using a 50 W Tungsten halogen lamp and deuterium interface with a quartz cuvette of a cell path length of 1cm. All measurements were taken in the wavelength range of 300-1100 nm. All the samples were freshly prepared before use. Data analyses were done using the inbuilt software version 2.34 and OriginPro version 8.5.

All the fluorescence emission studies were carried out using the Princeton Instrument-made Fluorescence Spectrometer SP-2150 instrument with external 808 (MDL-III-5W) semiconductor laser as the excitation source and imaged with a NIRvana ST 640 CCD Camera with ResXtreme and 1200 g/mm grating at 435.8 nm 20-micron pixel, 20-micron slit width.

UV-visible spectra were obtained for Ag_2S QD and compared with commercially available ICG. **Figure S1 (a)** shows that Ag_2S QD offers a broad excitation window ranging from 300 nm to 1100 nm. However, ICG shows a restricted excitation window ranging from 600 nm to 900 nm [see, **Figure S1 (c)**]. The emission spectra of ICG and QD upon identical 808nm excitations extends beyond 900nm, but ICG emission tails off rapidly >1000 nm, whereas QD emission peaks ~1250nm.

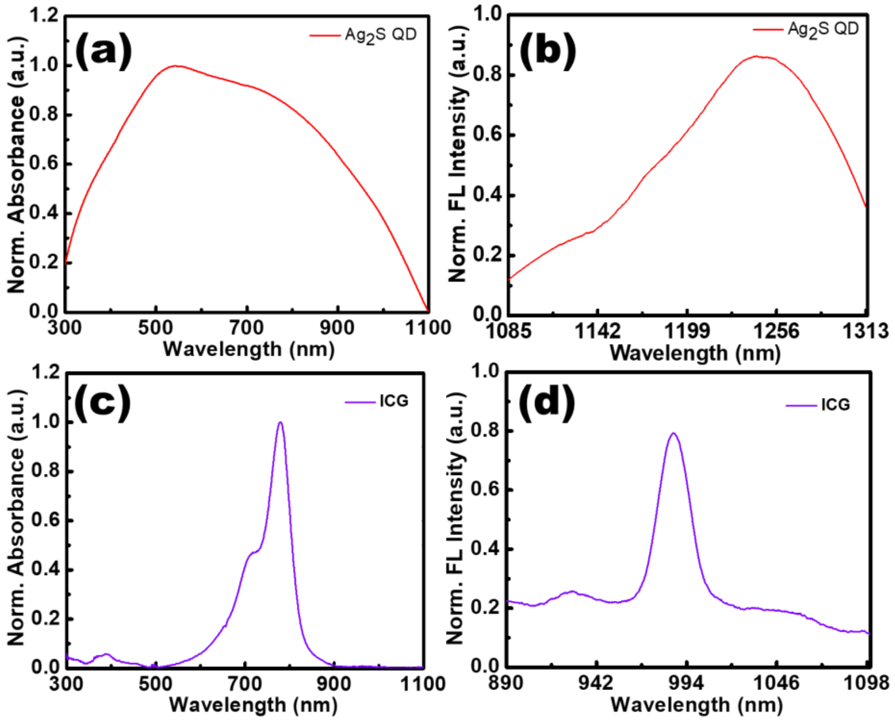

Figure S1. UV-Visible and FL Spectra of synthesized Ag_2S QD (a)-(b) and ICG (c)-(d)
